# The Potential for PE Microplastics to Affect the Removal of Carbamazepine Medical Pollutants from Aqueous Environments by Multiwalled Carbon Nanotubes

**DOI:** 10.3390/toxics9060139

**Published:** 2021-06-12

**Authors:** Xiaoyu Sheng, Junkai Wang, Wei Zhang, Qiting Zuo

**Affiliations:** 1 School of Ecology and Environment, Zhengzhou University, 100 Kexue Avenue, Zhengzhou 450001, China; sxy15670527525@163.com (X.S.); 13014502079@163.com (J.W.); 2 Yellow River Institute for Ecological Protection & Regional Coordinated Development, Zhengzhou University, Zhengzhou 450001, China; 3 Henan International Joint Laboratory of Water Cycle Simulation and Environmental Protection, Zhengzhou 450001, China; 4 Zhengzhou Key Laboratory of Water Resource and Environment, Zhengzhou 450001, China; 5 Henan Province Key Laboratory of Water Pollution Control and Rehabilitation Technology, Pingdingshan 467036, China; 6 School of Water Conservancy Engineering, Zhengzhou University, 100 Kexue Avenue, Zhengzhou 450001, China

**Keywords:** PE microplastics, multiwalled carbon nanotubes, carbamazepine, adsorption, MCNTs−coated PE microplastics

## Abstract

Microplastics are ubiquitous in aquatic environments and interact with other kinds of pollutants, which affects the migration, transformation, and fate of those other pollutants. In this study, we employ carbamazepine (CBZ) as the contaminant to study the influence of polyethylene (PE) microplastics on the adsorption of CBZ pollutants by multiwalled carbon nanotubes (MCNTs) in aqueous solution. The adsorption capacity of CBZ by MCNTs in the presence of PE microplastics was obviously lower than that by MCNTs alone. The influencing factors, including the dose of microplastics, pH, and CBZ solution concentration, on the adsorption of CBZ by MCNTs and MCNTs−PE were thoroughly investigated. The adsorption rate of CBZ by MCNTs decreased from 97.4% to 90.6% as the PE microplastics dose increased from 2 g/L to 20 g/L. This decrease occurred because the MCNTs were coated on the surface of the PE microplastics, which further decreased the effective adsorption area of the MCNTs. This research provides a framework for revealing the effect of microplastics on the adsorption of pollutants by carbon materials in aqueous environments.

## 1. Introduction

Plastic is an essential part of our daily life, and is used in items such as packaging, plastic bottles, computers, and even complex items such as airplanes [1]. The global production of plastics increased from 1.3 million tons in 1950 to 359 million tons in 2018 [2]. Plastic pollution has aroused widespread concern and attention. Plastic particles are difficult to degrade by weathering in the natural environment, and they tend to accumulate in aquatic environments and persist for many years [3,4,5]. Microplastics pollution is already an urgent issue because organisms can uptake the microplastics, which accumulate in the biological body, eventually entering other organisms through the food chain [6,7]. Microplastics are also considered carriers of pollutants due to their small size and large specific surface area [8,9], and they have a relatively high affinity for toxic chemicals such as pharmaceuticals and personal care products (PPCPs) [10] and heavy metals [11,12,13]. An increasing number of studies have been conducted on the interactions and interfacial adsorption processes between microplastics and other pollutants in different media environments (water and soil), but few studies have focused on the influence of the presence of microplastics on these interactions.

Carbon nanomaterials are used in a wide variety of fields, such as the manufacturing of automotive and electrical/electronic goods, renewable energy, sports, and the pharmaceutical industry [14]. The commercial production of one type of carbon material [15], carbon nanotubes (CNTs) [16], is growing rapidly, which inevitably results in a large quantity of CNT particles entering the wastewater environment, potentially affecting the transportation of other chemicals such as heavy metals [17,18], organic pollutants and antibiotics [19,20]. CNTs are reported to have a porous structure, a large specific surface area and abundant surface functional groups (O−H, C=C), which have relatively strong adsorption characteristics for sulfamethazine, carbamazepine, dimethyl phthalate and oxytetracycline [21,22,23,24]. However, the interaction between CNTs and pollutants is usually affected by other materials in the natural water environment, such as the pH, water flow, and other emerging pollutants. CNTs might exhibit strong affinity to different types of microplastics in experimental laboratory water and natural wastewater [10]. Thus, it is of interest to investigate the potential effect of microplastics on the interaction of CNTs with pollutants (such as medical pollutants) in aqueous solutions.

As reported, previous studies [25,26,27] have mainly emphasized the adsorption performance of emerging pollutants by some kinds of adsorbents in aqueous solutions. However, very few studies have focused on the influence of MPs on the removal of emerging contaminants from aqueous environments by adsorbents. Thus, it is necessary to explore the interfacial interaction between MPs and other adsorbents in aqueous environments and its influence on the migration and transformation of pollutants in an aqueous solution. To our knowledge, the adsorption performance of carbamazepine by CNTs in the presence of MPs has not yet been explored. Moreover, the function of the interface between MCNTs and PE microplastics urgently needs to be studied. This study is also valuable for evaluating the potential risk of MPs that affect the removal of emerging contaminants by carbon materials in aquatic environments.

## 2. Materials and Methods

### 2.1. Materials

Carbamazepine (CBZ, purity >99%) was purchased from Shanghai Macklin Biochemical Co., Ltd., Shanghai, China. The fundamental physical and chemical properties of CBZ were given in the Supplementary Materials, Table S1. PE microplastics (PE < 300 µm [28]) were purchased from Guangzhou Bofeng Chemical Technology Co., Ltd., Guangzhou, Guangdong, China. The MCNT_S_ (outer diameter of 50 nm, inner diameter of 20–30 nm, and length of 10–30 µm) were purchased from Suzhou Tanfeng Graphene Technology Co., Ltd., Suzhou, Jiangsu, China. The other reagents used in this experiment, including sodium hydroxide (NaOH) and concentrated sulfuric acid (H_2_SO_4_) of analytical reagent grade, were supplied by Tianjin Hengxing Chemical Reagent Manufacturing Co., Ltd., Tianjin, China and Luoyang Chemical Reagent Factory, Luoyang, Henan, China, respectively.

### 2.2. Adsorption Experiments

The batch adsorption experiments for MCNT_S_ and MCNT_S_−coated PE (MCNT−PE) were both conducted in 100 mL conical flasks covered with a sealing membrane to avoid solution evaporation. The samples were divided into three parallel groups, and all experiments were repeated three times. The flow chart for the whole experiment was shown in the Supplementary Materials, Figure S1. The MCNT_S_ were mixed with CBZ solution in 100 mL conical flasks and then oscillated at a speed of 150 oscillations/min at 298 K. After a certain time, the CBZ solution and adsorbent were separated with 0.22 µm miniature disposable needles. The initial and final adsorptions of CBZ were measured by a UV759CRT spectrophotometer (Shanghai Yoke Instrument Co., Ltd., Shanghai, China) at wavelengths of 285 nm; these measurements were then used to calculate the CBZ concentrations. The control experiment showed that the loss of CBZ during the adsorption process was negligible. The extent of adsorption and the percent removal efficiency (R) were evaluated according to Equations (1) and (2).

(1) $$q_{t}=\frac{\left(c_{0}-c\right)V}{m}$$

(2) $$R=\frac{\left(c_{0}-c\right)\times 100\%}{c}$$

In the equations, *m* (g) is the amount of the adsorbent in the CBZ solution. *V* (L) is the volume of the CBZ solution. *c*_0_ (mg/L) and *c* (mg/L) represent the initial and equilibrium concentrations of CBZ after 24 h of shaking, respectively.

#### 2.2.1. Effect of PE Microplastics Addition

The effect of PE microplastics doses on CBZ adsorption by 0.8 g/L MCNT_S_ was investigated by adding different amounts of PE microplastics (2, 4, 8, 12, 16, and 20 g/L) with an initial CBZ concentration of 10 mg/L for 24 h and an oscillation speed of 150 oscillations/min at 298 K.

#### 2.2.2. Effect of Contact Time

To analyze the influence of contact time on the CBZ adsorption by MCNT_S_ with and without PE microplastics, 0.8 g/L MCNT_S_ were added to conical flasks, and 20 g/L PE microplastics were added to one of the flasks. Before the investigation of adsorption, a preliminary experiment on the adsorption efficiency of CBZ on different doses of MCNTs established an optimum MCNT_S_ dose; the results are presented in Supplementary Materials, Figure S2. The adsorption behavior was investigated by adding 0.8 g/L MCNT_S_ to 25 mL CBZ solution (10 mg/L), and oscillating at a speed of 150 oscillations/min under 298 K. The PE microplastics (20 g/L) were added to the solution under the same conditions to analyze the effect of the PE microplastics on the adsorption of CBZ by the MCNT_S_. Thirteen sampling time points were set (0.5, 1, 5, 10, 30, 60, 180, 300, 480, 720, 900, 1080, and 1440 min), and three parallel samples were selected from each sampling time point.

#### 2.2.3. Effect of Initial CBZ Concentration

Because our study focused on the adsorption mechanism, the initial concentration of CBZ was set up along a gradient of five concentrations (10, 20, 30, 40 and 50 mg/L) in the isothermal adsorption experiments; this design was in keeping with that of a previous study [29,30]. First, 0.8 g/L of MCNT_S_ were added to each conical flask. Next, 25 mL CBZ solution along the concentration gradient was added to a different flask containing the MCNTs. The conical flasks were oscillated for 24 h at 298 K and a speed of 150 oscillations/min. Other experimental conditions remained unchanged, and each analysis of each concentration was performed in three parallel groups. On this basis, 0.8 g/L MCNT_S_ and 20 g/L PE microplastics were added to the solutions under the same conditions to analyze the effect of PE microplastics.

#### 2.2.4. Effect of pH

The effect of pH on the adsorption behaviors of CBZ on 0.8 g/L MCNT_S_ and 20 g/L PE microplastics was determined. The pH range of the CBZ solution was adjusted to 3, 5, 7, 9, and 11 using 0.1, 0.5, 1 mol/L NaOH and H_2_SO_4_ solutions, respectively, in 25 mL CBZ solution (10 mg/L) for 24 h. The experimental conditions were the same as those mentioned above.

### 2.3. Adsorption Model Investigation

A series of models have been developed to describe the adsorption kinetics to estimate the equilibrium time and predict its adsorption rate [25]. The pseudo−first−order and pseudo−second−order kinetic models were used to acquire the details of the adsorption kinetics of CBZ onto the adsorbents, as shown in Equations (3) and (4), respectively.

(3) $$\mathrm{Pseudo}-\mathrm{first}-\mathrm{order}\;\mathrm{kinetic}\;\mathrm{model}:\;\ln\left(q_{e}-q_{t}\right)=\ln q_{e}-k_{1}t$$

(4) $$\mathrm{Pseudo}-\sec\mathrm{ond}-\mathrm{order}\;\mathrm{kinetic}\;\mathrm{model}:\;\frac{t}{q_{t}}=\frac{1}{k_{2}q_{e}^{2}}+\frac{t}{q_{e}}$$

where *q*_*e*_ (mg/g) is the amount of pollutant adsorbed at equilibrium (mg) per gram of absorbent (g); *q*_*t*_ (mg/g) is the amount of adsorbed pollutant per gram of absorbent at time *t* (min); *k*_1_ (min^−1^) and *k*_2_ (g/(mg·min)) represent the adsorption rate constants of pseudo−first−order and pseudo−second−order kinetic models, respectively.

The thermodynamic values were determined at 288 K, 298 K, 308 K and 318 K. The thermodynamic parameters such as the Gibbs free energy (ΔG), enthalpy (ΔH) and entropy (ΔS) of adsorption of CBZ by both MCNTs or MCNTs−PE were calculated using the following Equations (5)–(7):

(5) $$K=\frac{q_{e}}{c_{e}}$$

(6) ΔG=−RTlnK

(7) $$lnK=\frac{\Delta S}{R}-\frac{\Delta H}{RT}$$

where *R* is the universal gas constant (8.314 J/mol K); *K* (L/g) is the thermodynamic equilibrium constant and *T* (K) is the absolute temperature. The values of ΔH were calculated from the slope and ΔS from the intercept of the linear curve of ln *K* and 1/*T*.

**Figure 1:**
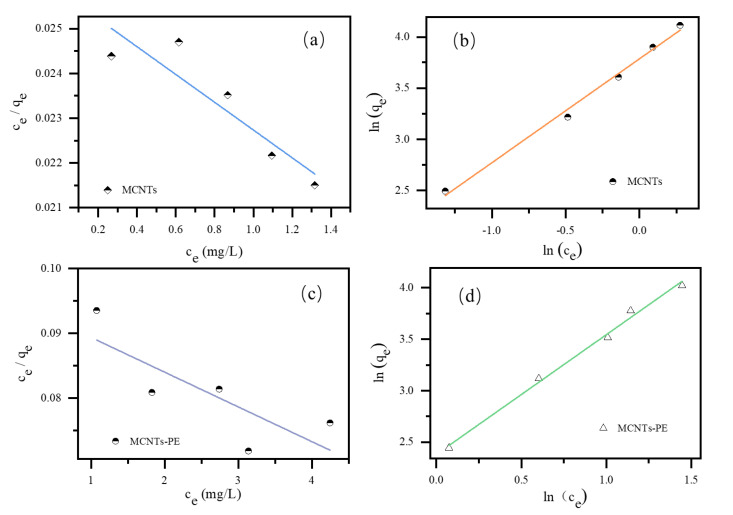
The adsorption isotherm fitting models: (**a**) Langmuir model of MCNTs on CBZ (by single MCNTs); (**b**) Freundlich model of MCNTs on CBZ (by single MCNTs); (**c**) Langmuir model of MCNTs−PE on CBZ (with PE added into MCNTs containing solution); (**d**) Freundlich model of MCNTs on CBZ (with PE added into MCNTs containing solution). (Conditions: the initial concentration 10, 20, 30, 40, 50 mg/L CBZ, MCNTs dose 0.8 g/L, PE dose 20 g/L and temperature 298 K).

Adsorption isotherm models were used to predict the distribution of CBZ at adsorption equilibrium between solid−phase concentration and aqueous−phase concentration between liquid and solid phases [31,32]. To further explore the adsorption mechanism of CBZ by the MCNTs, the adsorption isotherm study results for the CBZ adsorption process were shown in Figure 1 with the model fitting parameters summarized in the Supplementary Materials, Table S2. The adsorption isotherms were studied by the Langmuir and Freundlich models [33], which can be described by Equations (8) and (9), respectively.

(8) $$\mathrm{Langmuir}\;\mathrm{model}:\;\frac{c_{e}}{q_{e}}=\frac{c_{e}}{q_{max}}+\frac{1}{q_{max}b}$$

(9) $$\mathrm{Freundlich}\;\mathrm{model}:\;lnq_{e}=lnk_{F}+\frac{1}{n}lnc_{e}$$

where $k_{F}$ ([mg/g] [L/mg]^1/n^) is the Freundlich affinity coefficient; *n* is Freundlich heterogeneity factor; $c_{e}$ (mg/L) is the CBZ concentration at equilibrium; $q_{max}$ (mg/g) is the maximum adsorption capacity; *b* (L/mg) is the Langmuir adsorption equilibrium constant.

### 2.4. Material Characterization

A Gemini SEM 300 scanning electron microscope (SEM) was used to visualize the surface of the tested PE microplastics, MCNTs−PE and the agglomeration of MCNTs (Carl Zeiss AG, Oberkochen, Germany). Fourier transform infrared spectroscopy (FTIR) was used to analyze the functional groups of MCNTs in the region of 500–4000 cm^−1^ (TENSOR27, Bruker, Germany). The zeta potentials of the MCNTs and MCNT−PE samples in aqueous solution were measured using a JS94HM Zeta potentiometer (Shanghai Zhongchen Digital Technology Equipment Co., Ltd., Shanghai, China). A diffractometer was used for the X-ray diffraction (XRD) analysis (D/max 2500 pcX, Rigaku, Tokyo, Japan) of MCNTs before and after adsorption, MCNTs−PE and PE microplastics.

## 3. Results and Discussion

### 3.1. Effect of PE on the Adsorption of CBZ by MCNT_S_

**Figure 2:**
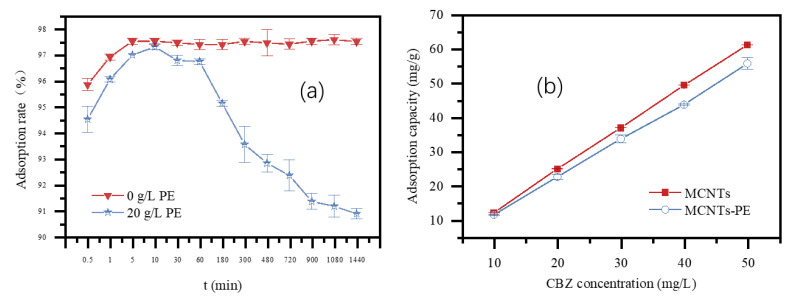
Effect of contact time (**a**) and CBZ concentration (**b**) on the adsorption of CBZ by MCNTs. (Conditions: the MCNTs dose was 0.8 g/L, the CBZ solution was 25 mL, and the contact temperature was 298 K).

The adsorption of CBZ by MCNT_S_ with and without the addition of PE microplastics was shown in Figure 2a. With single MCNT_S_, the adsorption of CBZ quickly reached equilibrium (adsorption rate of 97.5%) within 5 min and then remained nearly constant with increasing contact time for 24 h. Likewise, for the adsorption of CBZ by MCNTs in the presence of PE (20 g/L), the rate of the adsorption was similar that without the addition of PE within the first 5 min. However, the adsorption rate decreased gradually after 5 min in the presence of PE microplastics, with the adsorption rate decreasing to 90.9% at 24 h. Compared with the absence of PE microplastics, the adsorption rate was reduced by approximately 6.6%.

**Figure 3:**
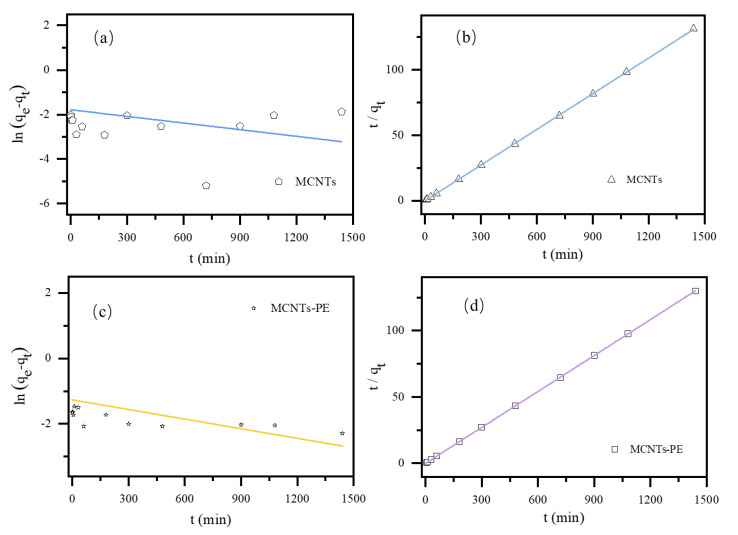
The adsorption kinetic model: (**a**) pseudo−first−order kinetic model of CBZ adsorbed by MCNTs (with only MCNTs); (**b**) pseudo−second−order kinetic model of CBZ adsorbed by MCNTs (with only MCNTs); (**c**) pseudo−first−order kinetic model of CBZ adsorbed by MCNTs−PE (with PE added into the solution containing MCNTs); (**d**) pseudo−second−order kinetic model of CBZ adsorbed by MCNTs−PE (with PE added into the solution containing MCNTs). (Conditions: CBZ solution of 25 mL, MCNTs dose of 0.8 g/L, PE dose of 20 g/L, and a contact temperature of 298 K).

The kinetic models of CBZ adsorption on MCNTs with and without PE microplastics were investigated and shown in Figure 3 and the Supplementary Materials, Table S3. The results indicated that CBZ adsorption by MCNTs and MCNTs−PE followed the pseudo−second−order model. This result suggested that it was not a monolayer physical adsorption process, further confirming that this process was mainly controlled by chemisorption [26,34], consistent with what Oleszczuk et al. proved in their experiment [21]. According to the kinetic models, the adsorption of CBZ on MCNTs after the addition of PE did not affect its compliance with the model. The FTIR spectra of MCNTs before and after adsorption as shown in Figure S3. It was speculated that the influence of PE microplastics on adsorption does not change the chemisorption of CBZ. 

The thermodynamic parameters for the adsorption of CBZ onto MCNTs and MCNTs−PE were listed in the Supplementary Materials, Tables S4 and S5, respectively. The enthalpy changes (ΔH) for the adsorption of CBZ onto the MCNTs and MCNTs−PE were −34.82 kJ/mol and −66.85 kJ/mol at four temperatures, indicating that the adsorption of CBZ by CNTs and CNTs−PE was an exothermic process in both cases. Lower temperatures were more conducive to the adsorption of CBZ by the MCNTs or MCNTs−PE. With increasing adsorption temperature, the surface tension of the MCNTs decreased, leading to decrease in the adsorption of CBZ on the MCNTs [35]. The negative value of ΔS demonstrated that the distribution of CBZ in aqueous solution was more chaotic. This result revealed the disorder at the solid/solution interface increased during the adsorption of CBZ on the MCNTs and MCNTs−PE. The temperature ranged from 288 K to 318 K, and the ΔG values of the adsorption of CBZ onto the MCNTs were −11.23 kJ/mol, −9.90 kJ/mol, −9.56 kJ/mol and −8.61 kJ/mol. The ΔG values for the adsorption of CBZ on the MCNTs−PE were −7.10 kJ/mol, −5.87 kJ/mol, −3.09 kJ/mol and −1.11 kJ/mol. The negative values of ΔG indicated that the adsorption process was spontaneous and that CBZ was more easily adsorbed from the solution to the surface of the MCNTs and MCNTs−PE.

Figure 2b presented the adsorption capacity of CBZ by MCNTs with and without PE microplastics at varying initial CBZ concentrations. Comparing the adsorption mass of CBZ by MCNTs and MCNTs−PE showed that more CBZ pollutants were adsorbed by single MCNTs than MCNTs−PE. The adsorption capacity of MCNTs and MCNTs−PE both increased with an increase in the initial CBZ solution concentration. Due to the concentration gradient, the equilibrium adsorption capacity of CBZ by MCNTs and MCNTs−PE also increased with increasing CBZ concentration, which was the driving force to overcome resistance to the mass transfer of organic compounds between the aqueous and solid phases [36]. A higher initial concentration provides strong mass transfer power, resulting in a higher adsorption capacity.

The Freundlich model better describes the experimental data (R^2^ > 0.99) than the Langmuir model, which could not be used to fit the experimental data, as shown in Table S2. This result could indicate that the adsorption process of CBZ was mainly multilayer adsorption on the heterogeneous surface of MCNTs or MCNTS−PE [37]. According to the Freundlich model, the amount of adsorbed CBZ increased infinitely with increasing initial CBZ concentrations. Thus, the saturation adsorption amount could not be predicted only by the Freundlich isotherm model [25,38]. The values of K_F_ reflected the maximum adsorption capacity of CBZ by the CNTs and CNTs−PE, indicating that the maximum adsorption capacities of CBZ by MCNTs and MCNTs−PE were 10.29 and 6.47 [mg/g] [L/mg]^1/n^, respectively. These values also indicated that the addition of PE microplastics to the aqueous solution inhibited the adsorption of CBZ by the MCNTs.

### 3.2. Effect of Varying the PE Microplastics Dose

**Figure 4:**
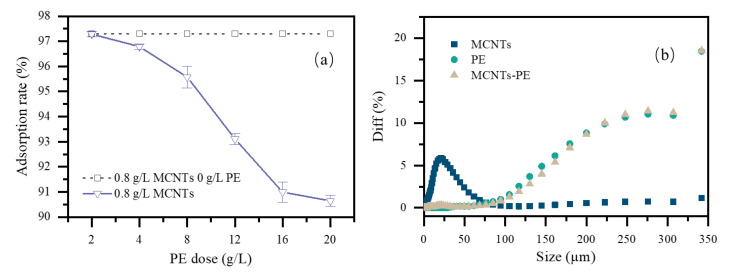
The effect of PE microplastic doses and hydrodynamic diameter distribution of PE microplastic, MCNTs, and the MCNTs−PE: (**a**) the adsorption rate of CBZ by the 0.8 g/L MCNTs from a 25 mL CBZ solution (10 mg/L) at an initial pH of 7 for 24 h under 298 K; (**b**) hydrodynamic diameter distribution of PE microplastic, MCNTs, and the MCNTs−PE under 298 K.

**Figure 5:**
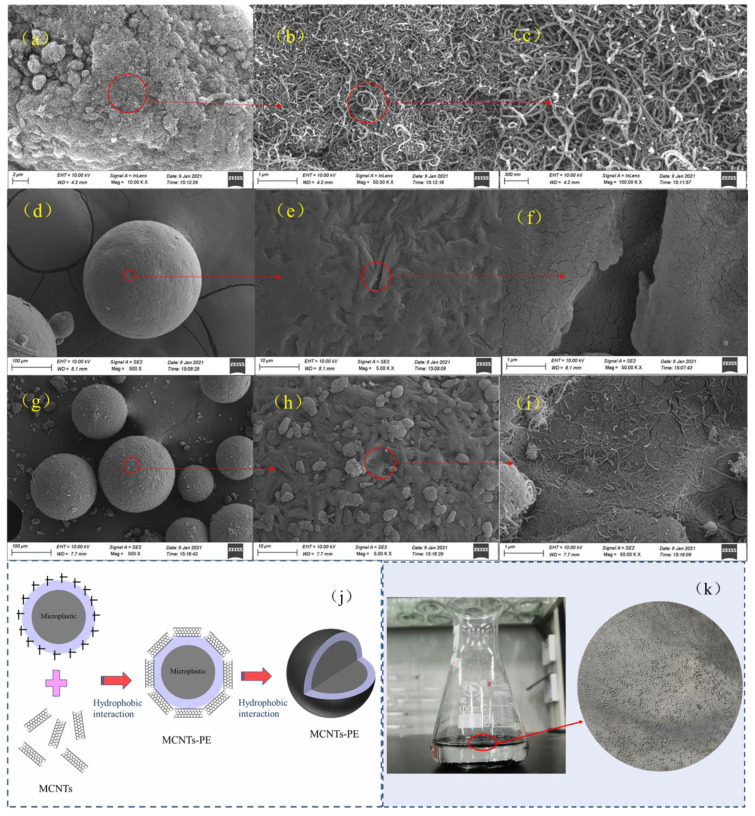
SEM images of the MCNTs, PE microplastic, MCNTs−PE and the mechanism and phenomenon diagram of MCNTs−coated PE: (**a**–**c**) at magnification × 10 k, 50 k, 100 k; SEM images of PE microplastic (**d**–**f**) at magnification × 500, 5 k, 50 k; SEM images of MCNTs−PE (**g**–**i**) at magnification × 500, 5 k, 50 k, respectively. (**j**) The mechanism diagram of MCNT−coated PE; (**k**) the phenomenon diagram of MCNT−coated PE.

Figure 4a presented the influence of increasing the PE microplastics dose on the adsorption rate of CBZ by single MCNTs (0.8 g/L). When the amount of PE microplastics was 2 g/L, the adsorption rate of CBZ by MCNTs was close to 97.3%. When the PE dose was further increased to 4 g/L, the adsorption rate of CBZ decreased from 97.4% to 96.7% at an MCNT dose of 0.8 g/L. At PE microplastics dose of 20 g/L, the adsorption rate of CBZ by the MCNTs dropped to 90.6%; this drop was significant compared with the 97.3% adsorption rate at PE microplastics dose of 2 g/L. The obvious decrease in the adsorption rate was mainly attributed to the reduction in adsorption sites due to the MCNT surfaces being coated with PE microplastics (MCNTs−PE), as shown in Figure 5. The surfaces of the original PE microplastics were relatively smooth spheres, but they became rougher after being coated with the tubular MCNTs. The MCNTs aggregated on the surface of the PE microplastics. XRD spectra of CNTs and CNTs−PE after the adsorption of CBZ (Figure S3) evidenced a small amount of CBZ adsorption did not affect the crystallinity of MCNTs and CNTs−PE. The mechanism diagram and photograph of the appearance of the MCNTs coating on the PE microplastics were shown in Figure 5j,k, respectively. Tang et al. [10] observed similar results when removing microplastics from aqueous solutions by magnetic carbon nanotubes. The hydrodynamic diameter analysis results (Figure 4b) of the MCNTs, PE microplastics, and MCNTs−PE in aqueous solution demonstrated that the hydrodynamic diameter of the MCNTs was approximately 19.5 µm due to their own agglomeration, as shown in the SEM images (Figure 5a–c), while the average particle sizes for PE and MCNTs−PE were approximately 224.9 µm and 227.4 µm, respectively.

### 3.3. Effect of pH

**Figure 6:**
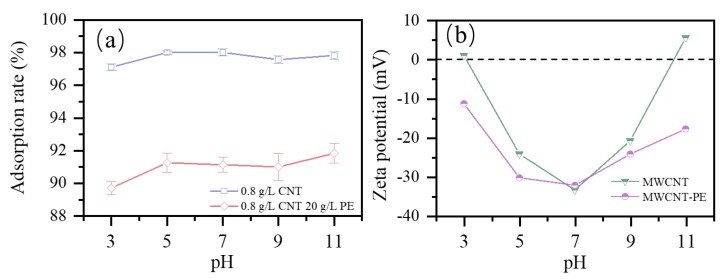
Effect of pH and Zeta potential: (**a**) effect of pH on the adsorption of CBZ (25 mL of 10 mg/L CBZ solution, MCNTs dose of 0.8 g/L, PE dose of 20 g/L and temperature of 298 K); (**b**) zeta potential of MCNTs and MCNTs−PE at different pH.

The effect of pH on CBZ adsorption by the MCNTs with the addition of PE microplastics was shown in Figure 6a. Zeta potentials of MCNTs and MCNTs−PE at different pH were shown in Figure 6b. CBZ can be well adsorbed in a wide pH range, regardless of the surface charging of the MCNTs and MCNTs−PE, and these results were also reported in [39]. The adsorption rate of CBZ by both MCNTs and MCNTs−PE changed by less than 1% at different pH values. CBZ molecules were present in hydrophobic zwitterionic forms in the investigated pH range (3 to 11) [29]. MCNTs can adsorb different types of micropollutants (neutral and ionic) in aqueous environments, and there was no significant difference in the adsorption affinity of CNTs for neutral compounds [40]. The electrostatic interaction of MCNTs and MCNTs−PE adsorption on CBZ was negligible. This is consistent with the results of previous studies. Yan [39] et al. investigated the ability of carbon dot−modified magnetic carbon nanotubes to maintain the ability to adsorb CBZ well over the whole pH range. Ncibi [29] et al. demonstrated that the electrostatic interaction could be neglected for the adsorption of CBZ by mesoporous activated carbons and multi−walled carbon nanotubes. The hydrophobic and π–π interactions between CBZ and MCNTs (or MCNTs−PE) should also be considered. Compared with MCNTs−PE, more CBZ was adsorbed on MCNTs only in the pH range of 3 to 11. The addition of PE reduced the adsorption rate at different pH values, indicating that pH values do not affect the coating structure of MCNTs−PE.

### 3.4. Mechanism of CBZ Adsorption by MCNTs and the Influence of PE Microplastics

The interaction mechanism of organic pollutants and MCNTs is mainly related to physical adsorption and chemical adsorption [24,41]. The pseudo−second−order kinetic model and the Freundlich models obtained from the study confirm that the adsorption of CBZ by MCNTs was mainly controlled by the chemisorption process. Electrostatic interactions were not dominant because CBZ was neutral molecule [42] in the investigated pH range from 3 to 11. The hydrophobic interaction was dominant between CBZ (with hydrophobic and nonpolar characteristics [29]) and the MCNTs (with hydrophobic surfaces [43]). Thus, the hydrophobic surfaces of MCNTs lead to a higher removal efficiency of CBZ. Moreover, π–π electron donor–acceptor (EDA) interactions also participate in the adsorption of CBZ by CNTs. CBZ was expected to act as a π−electron acceptor due to the electron withdrawing capability of the amide group on the CBZ, which could form π–π EDA interactions with the surface of the MCNTs [44,45]. In summary, the adsorption mechanism of CBZ onto MCNTs mainly includes hydrophobic interactions and π–π interactions during the CBZ chemisorption process.

**Figure 7:**
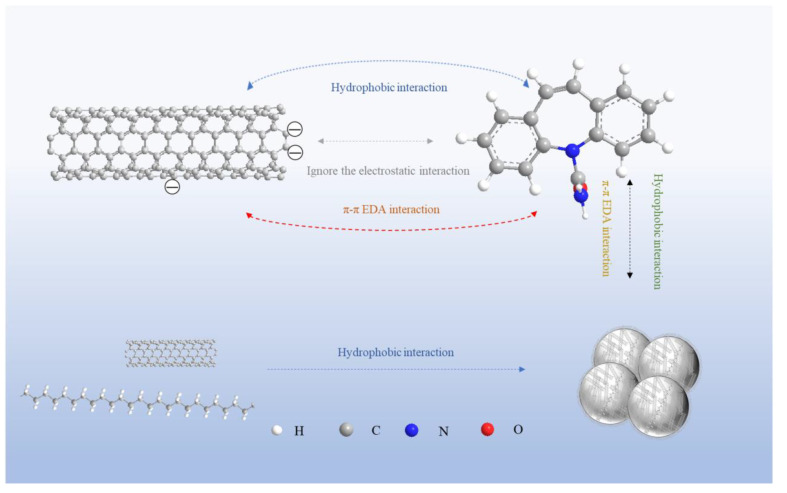
The proposed adsorption mechanism for PE affecting the adsorption of CBZ by MCNTs and interaction mechanism between MCNTs and PE.

As shown in Figure 6a, the adsorption of CBZ by MCNTs was not significantly changed after adding PE microplastics at different pH values, indicating that the MCNTs had little influence on the electrostatic interaction due to CBZ adsorption. The added PE microplastics did not directly affect the π–π EDA interactions between the MCNTs and CBZ because the addition of PE microplastics did not damage the MCNTs’ surface structure (which is rich in free π electrons as π−electron donors) and CBZ (which acts as a π−electron acceptor). The observed coating phenomenon of MCNTs on PE microplastics (Figure 5) demonstrated that MCNTs had a strong affinity for MPs (with different surface properties for various kinds of MPs), and MCNT coating of the PE was mainly attributed to the strong hydrophobicity of the MPs [10]. With the addition of PE microplastics, the surface of PE microplastic particles were coated by carbon nanotubes to form MCNT−PE composite particles that did not easily separate in the CBZ solution (Supplementary Materials, Figure S2b). After the MCNTs coated the surface of the PE microplastics, the adsorption area for MCNTs decreased, and the number of single CNTs in the aqueous solution also decreased after coating the surface of MPs, resulting in a reduction in the adsorption rate of CBZ by MCNTs (as described in Section 3.2.). Due to the hydrophobic interactions of MCNTs and PE microplastics between the water molecules, the MCNTs−PE composite particles further agglomerate, which further reduces the effective absorption area of the MCNTs. The proposed adsorption mechanism for how PE affects the adsorption of CBZ by MCNTs was illustrated in Figure 7.

## 4. Conclusions

In this study, the effect of PE microplastics on the adsorption of CBZ from an aqueous solution was analyzed based on an investigation of CBZ adsorption by MCNTs. This study further analyzed the effects of microplastics with various properties on the adsorption of CBZ by MCNTs.

(1) The results showed that the removal of CBZ on MCNTs was inhibited in the presence of PE microplastics. When the dose of PE microplastics was 2 g/L, the adsorption rate of CBZ by MCNTs was 97.3% of the rate of adsorption without PE microplastics; this dose had little effect on CBZ adsorption by the MCNTs. However, with increasing PE microplastics dosage, the inhibitory effect of MCNTs on CBZ adsorption became more obvious. When 20 g/L PE was added, the adsorption rate of CBZ by MCNTs was 6.76% lower than that by MCNTs only.

(2) The experimental and theoretical results showed that hydrophobic interactions and π–π interactions were the main mechanisms of adsorption on CBZ by MCNTs and MCNTs−PE. The mechanism of MCNTs coating the PE microplastics mainly involves hydrophobic interactions. MCNTs and PE microplastics collide violently to form MCNT−PE composite particles that do not easily separate in the CBZ solution. After the MCNTs coated the PE microplastics, the effective adsorption area of the MCNTs decreased, leading to a decrease in the adsorption rate of CBZ by the MCNTs.

(3) In this study, only one kind of microplastic was used to influence the adsorption of pollutants by CNTs. Therefore, it is necessary to systematically study the interaction mechanism between other microplastics and CNTs, such as the interactions between polar and nonpolar microplastics and CNTs, and to further explore the interaction mechanism between substances in ternary systems.

## Data Availability

Not applicable.

## Supplementary Materials

The following are available online at https://www.mdpi.com/article/10.3390/toxics9060139/s1, Figure S1: Proposed experimental flowchart of a, b, c, d: (a) Effect of contact time; (b) Effect of initial CBZ concentration; (c) Effect of PE dosage; (d) Effect of pH. Figure S2: The influence of MCNTs dosage and comparison of MCNTs, PE and MCNTs−PE: (a) adsorption rate and capacity of CBZ by MCNTs dosage; (b) the surface of PE microplastic particles were coated by MCNTs to form MCNTs−PE composite particles. Figure S3: FTIR spectra and XRD patterns: (a) FTIR spectra of 10 mg/L 25 mL CBZ solution before and after adsorption on 0.8 g/L MCNTs at an initial pH of 7 for 24 h at a temperature of 298 K; (b) XRD patterns of the MCNTs, MCNTs after adsorption, PE and MCNTs−PE. Table S1: Physicochemical parameters of carbamazepine. Table S2: Langmuir model and Freundlich model fitting parameter table. Table S3: Adsorption rate constants for two kinetic models. Table S4: Thermodynamic parameters for the adsorption of CBZ onto MCNTs. Table S5: Thermodynamic parameters for the adsorption of CBZ onto MCNTs−PE [46,47].

## Abbreviations

MCNTs	Multiwalled carbon nanotubes
PE	Polyethylene microplastics
CBZ	Carbamazepine
MCNTs−PE	Multiwalled carbon nanotube−coated polyethylene microplastics
MPs	Microplastics
CNTs	Carbon nanotubes
K_F_	The constant of the equilibrium adsorption
SEM	Scanning electron microscope
XRD	X-ray diffraction
FTIR	Fourier transform infrared spectrometer
