# Supplementary material for: The Potential for PE Microplastics to Affect the Removal of Carbamazepine Medical Pollutants from Aqueous Environments by Multiwalled Carbon Nanotubes
Source: Toxics. 2021 Jun 12;9(6):139. doi: 10.3390/toxics9060139 (PMC8231597; doi:10.3390/toxics9060139)
Supplement: Supplementary file 1 [file toxics-09-00139-s001.zip › toxics-1247947-supplementary.pdf]

toxics

MDPI

Article

Supplementary Material: The Potential for PE Microplastics to Affect the Removal of Carbamazepine Medical Pollutants from Aqueous Environments by Multiwalled Carbon Nanotubes

Xiaoyu Sheng, Junkai Wang, Wei Zhang and Qiting Zuo

Table S1. Physicochemical parameters of carbamazepine.

Structure	Molecular Formula	MW (g/moL)	pKa1	pKa2	CAS No
$C_{15}H_{12}N_2O$	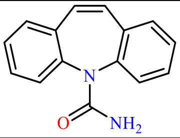	236.09	2.3	13.9	298-46-4

Table S2. Langmuir model and Freundlich model fitting parameter table.

Samples	Langmuir Model			Freundlich Model		
	b	q_{max}	R^2	n^{-1}	K_F	R^2
MCNTs	−0.12	−322.58	0.78	0.98	10.29	0.99
MCNTs−PE	−0.56	−186.22	0.52	0.86	6.47	0.99

Table S3. Adsorption rate constants for two kinetic models.

Samples	Pseudo−First−Order Kinetic Model		Pseudo−Second−Order Kinetic Model	
	k_1	R^2	k_2	R^2
MCNTs	0.001	0.07	−0.1	0.99
MCNTs−PE	0.06	0.13	23.2	0.99

Table S4. Thermodynamic parameters for the adsorption of CBZ onto MCNTs.

T (K)	ΔH (kJ/mol)	ΔS(J/mol·K)	ΔG (kJ/mol)
288	−34.82	−82.49	−11.23
298			−9.90
308			−9.56
318			−8.61

Table S5. Thermodynamic parameters for the adsorption of CBZ onto MCNTs−PE.

T (K)	ΔH (kJ/mol)	ΔS (J/mol·K)	ΔG (kJ/mol)
288	−66.85	−206.46	−7.10
298			−5.87
308			−3.09
318			−1.11

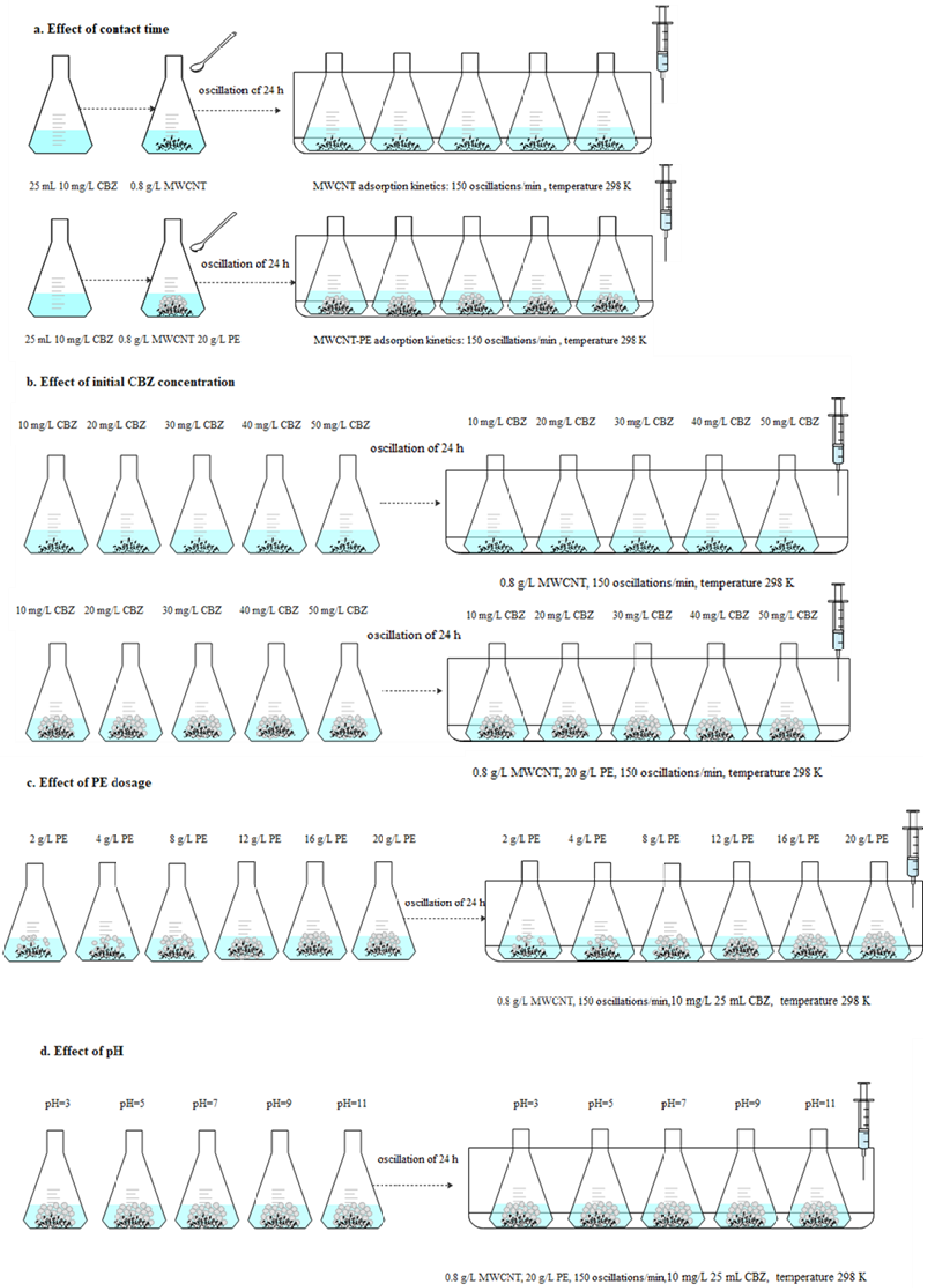

Figure S1. Proposed experimental flowchart of a, b, c, d. (a) Effect of contact time; (b) Effect of initial CBZ concentration; (c) Effect of PE dosage; (d) Effect of pH.

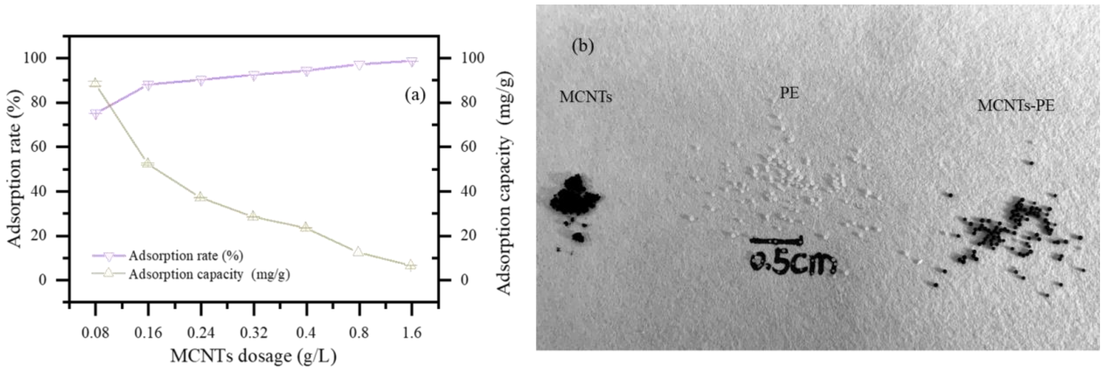

Figure S2. The influence of MCNTs dosage and comparison of MCNTs, PE and MCNTs−PE: (**a**) adsorption rate and capacity of CBZ by MCNTs dosage; (**b**) the surface of PE microplastic particles were coated by MCNTs to form MCNTs−PE composite particles.

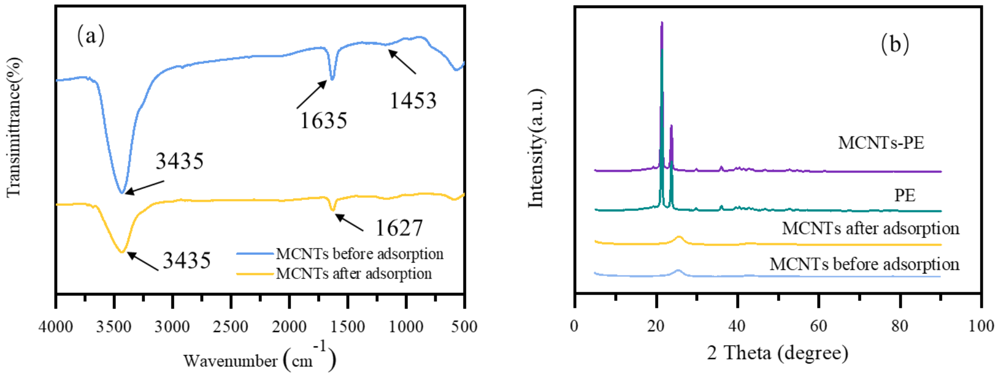

Figure S3. FTIR spectra and XRD patterns: (**a**) FTIR spectra of 10 mg/L 25 mL CBZ solution before and after adsorption on 0.8 g/L MCNTs at an initial pH of 7 for 24 h at a temperature of 298 K; (**b**) XRD patterns of the MCNTs, MCNTs after adsorption, PE and MCNTs−PE.

MCNTs exhibits the characteristic absorption peaks at 3430 cm^{-1}, which is attributed to the presence of O−H stretching [1]. The peak at 1635 cm^{-1} and 1590–1428 cm^{-1} corresponds to the C double bond O stretching mode and C double bond C stretching mode. 3464 and 1678 cm^{-1} correspond to the stretching of single bond N single bond H and C double bond O in CBZ molecules, respectively [2]. As can be seen from the infrared spectrum, the characteristic peak of MCNTs shifted from 1635 to 1627 compared with that before adsorption, which may be caused by the adsorption of CBZ on MCNTs. After adsorption CBZ on MCNTs, the ratio of intensity at 3435 cm^{-1} and 1635 cm^{-1} reduces. On the other hand, the vibration intensity of functional groups was affected by the change of environmental conditions in the solution, resulting in the weakening of characteristic peak intensity of MCNTs after adsorption.

XRD patterns of the MCNTs, MCNTs after adsorption, PE and MCNTs−PE were shown in Figure S2b. The XRD patterns of MCNTs before and after adsorption showed little change, indicating that a small amount of CBZ adsorption did not affect the crystallinity of MCNTs. Compared with the XRD of the original PE, CNTs−coated PE did not affect the crystallinity of PE.

References

1. Lawal, I.A.; Lawal, M.M.; Akpotu, S.O.; Azeez, M.A.; Ndungu, P.; Moodley, B. Theoretical and experimental adsorption studies of sulfamethoxazole and ketoprofen on synthesized ionic liquids modified CNTs. *Ecotoxicol. Environ. Saf.* **2018**, *161*, 542–552. doi:10.1016/j.ecoenv.2018.06.019.

2. Chen, D.; Sun, H.; Wang, Y.; Quan, H.; Ruan, Z.; Ren, Z.; Luo, X. UiO−66 derived zirconia/porous carbon nanocomposites for efficient removal of carbamazepine and adsorption mechanism. *Appl. Surf. Sci.* **2020**, *507*, 145054, doi:10.1016/j.apsusc.2019.145054.
